# Use of Gypsum as a Preventive Measure for Strength Deterioration during Curing in Class F Fly Ash Geopolymer System

**DOI:** 10.3390/ma8063053

**Published:** 2015-05-29

**Authors:** Yubin Jun, Jae Eun Oh

**Affiliations:** School of Urban and Environmental Engineering, Ulsan National Institute of Science and Technology (UNIST), Ulsan Metropolitan City 689-798, Korea; E-Mail: ssjun97@gmail.com

**Keywords:** fly ash, geopolymer, gypsum, Na_2_SO_4_, strength deterioration, nanometer-crack

## Abstract

This study discusses strength deterioration during the curing process of fly ash geopolymer and the use of CaSO_4_·2H_2_O (gypsum) as a deterioration remedy when the ash was synthesized using a 10M NaOH and Na-silicate solution. The strength decline was mainly due to the widespread formation of nanometer-sized cracks that were related to excessive Na and Si concentrations at an early age. Use of 2 wt% CaSO_4_**·**2H_2_O resulted in the best measured strength by temporarily reducing Na and Si concentrations; Na was absorbed by SO_4_^2−^, up to 11% in the matrix within one day, and formed Na_2_SO_4_ (thenardite), which gradually dissolved over time, slowly releasing Na ions. However, more than 4% gypsum suppressed overall strength development because too many Na ions were locked into Na_2_SO_4_ and could not participate in geopolymerization. The addition of gypsum impeded glass dissolution and even halted the process when more than 4% gypsum was used.

## 1. Introduction

Geopolymer is formed by mixing amorphous aluminosilicate materials with an alkaline solution, producing materials with mechanical properties comparable to Portland cement. The mechanical properties of geopolymer are primarily determined by several factors, such as the quality of the aluminosilicate, type of alkaline activator, activator concentration, activator/binder ratio, curing temperature, or curing time. In particular, an alkaline activator is essential for producing geopolymer with superior qualities [1,2].

When solely using a NaOH solution for alkali-activation of fly ash, an optimal NaOH concentration is required for increased compressive strength, and suboptimum concentrations have shown detrimental effects on strength development [3,4,5]. When Na-silicate is used in combination with NaOH, the mixture provides better mechanical strength than when only NaOH is used [6,7]. Palomo *et al*. [6] found that samples activated with NaOH showed a porous matrix, while those activated with NaOH and Na-silicate solutions showed a denser matrix in the microstructural study. The weight ratio of NaOH and Na-silicate in solution also affects the degree of strength gained, and the optimal ratio varies with the type of fly ash; excessive addition of Na-silicate tends to lower the strength of the hardened geopolymer [3,8].

Multiple incidents of strength deterioration with curing time in hardened geopolymers have been reported in the literature [9,10,11,12]. Most of these cases were observed when amorphous aluminosilicate materials were activated with highly concentrated Na-silicate, or in combination with NaOH, under elevated curing temperatures [9,10,12] or ambient conditions [11], while the sole use of a NaOH solution resulted in significantly fewer incidents of strength deterioration [9]. Many of the earlier studies claimed that the strength deterioration with curing time was related to a loss of moisture and possible shrinkage caused by drying during the curing process [9,10,11] or a phase transition from amorphous into crystalline zeolitic phases [12]. However, the sources of the strength deterioration processes are still not fully understood.

Gypsum was used in a few earlier studies to improve the strength of fly ash based geopolymers when using NaOH and Na-silicate to activate the fly ash [4,8,13,14]. The studies observed that the addition of a small quantity of gypsum increased the material strength, but the addition of more than an optimal quantity reduced the material strength. Although the studies claimed that the strength improvement was due to the formation of more C-S-H [8] or a higher degree of Al or Si dissolution from the fly ash [13,14], the claims were not confirmed. No sufficient explanation for the reduced strengths when the gypsum concentration exceeded certain limits was provided. Thus, the effect of gypsum as a chemical additive needs a more in-depth analysis.

This study provides a detailed discussion on the following two issues: (1) the strength deterioration observed in hardened geopolymers during the curing process when synthesized using a high concentration of Na-silicate in a NaOH solution and (2) the role of Na_2_SO_4_ synthesized from the addition of CaSO_4_·H_2_O, which is proposed as a potential remedy for the strength decline. This study was performed using compressive strength testing, X-ray diffraction (XRD), ultra-high resolution field emission scanning electron microscopy (FE-SEM) with energy dispersive spectroscopy (EDS), and mercury intrusion porosimetry (MIP) to support the discussion.

## 2. Experimental Section

### 2.1. Materials

The raw fly ash used in this study was sourced from a coal-fired power plant in Gyeongnam Province, South Korea. The chemical composition of the fly ash, determined by X-ray fluorescence (XRF), is given in Table 1. The weight sums of the SiO_2_, Al_2_O_3_, and Fe_2_O_3_ contents were greater than 70%, and thus, the fly ash used in this study was classified as Class F according to ASTM C 618. The fly ash was used as the main component for producing the target geopolymer. Calcium sulfate dihydrate (CaSO_4_·2H_2_O, gypsum) was used as an additive. Sodium hydroxide (NaOH) and Na-silicate solutions were used for alkaline activation. Gypsum (reagent grade, ≥99% assay), NaOH (reagent grade, ≥98% assay in pellet form) and the Na-silicate solution (reagent grade, 10.6% Na_2_O, 26.5% SiO_2_, and 62.9% H_2_O) were acquired from Sigma-Aldrich.

**Table 1:** Chemical composition (reported as oxide wt%) of fly ash.

SiO_2_	Al_2_O_3_	CaO	Na_2_O	K_2_O	MgO	MnO	TiO_2_	SO_3_	P_2_O_5_	Fe_2_O_3_	Others
51.8	20.0	10.1	0.6	1.0	2.0	0.1	1.2	0.9	1.4	10.3	0.6

### 2.2. Experimental Design and Sample Preparation

The mixture proportions of samples are tabulated in Table 2, where each sample is denoted with a specific letter, “F”, “G”, “N” and “W” representing the fly ash, gypsum, NaOH and Na-silicate solution, respectively. Fly ash was replaced with 2%, 4% and 6% gypsum by weight, denoted with “2”, “4” and “6” in the sample. The alkaline activator was prepared by blending 10M NaOH solutions with Na-silicate solutions. The mass ratio of the NaOH solution to the Na-silicate solution was 1.0. The 10M NaOH solution was prepared by dissolving NaOH pellets in de-ionized water. The weight ratio of the alkali activator (10M NaOH + Na-silicate) to the binder (FA + gypsum) was 0.4.

**Table 2:** Mixture proportions of samples.

Sample ID	Binder	Activator	Activator/binder (wt./wt.)	Curing temp. (°C)
	Fly ash(F):gypsum(G) (wt.:wt.)	10M NaOH(N)/Na-silicate solution(W) (wt./wt.)		
F-NW	100:0	1.0	0.4	60
FG2-NW	98:2			
FG4-NW	96:4			
FG6-NW	94:6			

The binder and alkali activator were hand-mixed for 5 min. After mixing, each paste was cast in three polyethylene cylinder molds with dimensions of 25.4-mm diameter × 25.4-mm height. For sample F-NW, to ensure the reproducibility of the strength deterioration measurements, sample sets were separately made and tested twice in unit blocks of three samples using the same fly ash, with a time interval of 10 days between each set (*i.e.*, six samples were made and tested in total). All of the samples were cured at a temperature of 60 °C with 99% relative humidity until the test period.

Compressive strengths of the hardened samples were measured at one, seven, and 28 days. Fractured specimens from the compression test were powdered and examined by XRD. To stop further hydration, the power samples for XRD test were solvent-changed with acetone and dried out in a vacuum desiccator [15]. The XRD patterns were collected on a Rigaku high power X-ray diffractometer using Cu Kα radiation. A scanning speed of 1°/min was used. The XRD patterns were analyzed using X’pert HighScore Plus software [16] with the ICDD PDF-2 database [17]. The SEM test was carried out on the samples cured for 28 days. The MIP test for representative samples was performed on the samples cured for one, seven and 28 days. Hardened samples were investigated using ultra-high resolution field emission scanning electron microscopy (FE-SEM, Hitachi S-4800, Hitachi, Tokyo, Japan) with energy dispersive spectroscopy (EDS) and mercury intrusion porosimetry (MIP, Autopore IV, Micrometrics, Atlanta, GA, USA), which was capable of providing a maximum mercury pressure of 414 MPa. To carry out the tests, specimens with a thickness of 2 mm along the length of the cylindrical samples were prepared using a precision saw. The sliced specimens were immersed in isopropanol to stop hydration. After vacuum drying, some of the samples were examined by MIP and the other equivalent samples were prepared for SEM analysis. The dried samples were put in a 25.4-mm diameter round mold to fit the SEM holder, impregnated with EPO-TEK epoxy resin under vacuum, and stored at an ambient temperature (23 ± 2 °C) for 24 h. For secondary electron (SE) images, the surfaces of the samples mounted in epoxy were cut off using a precision saw. For backscattered electron (BSE) images, sample surfaces were polished with a final grit of a 1/4 μm diamond polishing compound using an automatic grinder (EcoMet 250 Grinder-polisher, Buehler, Lake Bluff, IL, USA). The cut and polished sections were coated with a thin film of osmium before SEM observation.

## 3. Results and Discussions

**Figure 1:**
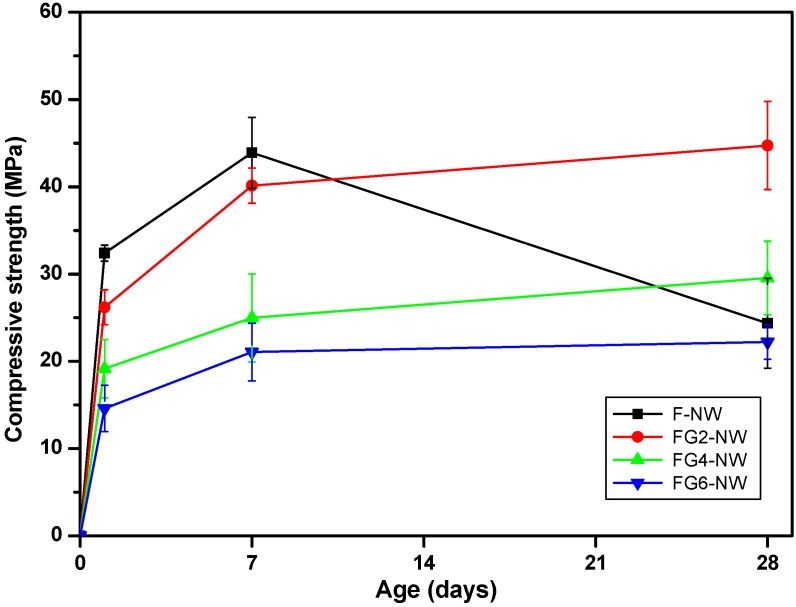
Compressive strength testing results of the samples containing different replacement ratios of gypsum. The labels “F”, “G”, “N” and “W” represent the fly ash, gypsum, NaOH and Na-silicate solution. F-NW: case of no gypsum addition; FG2-NW: 2% gypsum addition; FG4-NW: 4% gypsum addition; FG6-NW: 6% gypsum addition.

The compressive strength testing results of the samples containing different replacement ratios of gypsum are shown in Table 3 and Figure 1. Note that sample F-NW was made within a typical range of successful synthesizing conditions of activator and curing temperature for fly ash geopolymer, as reported in the literature [3,4,6,8,14,18]. Nonetheless, although the F-NW sample showed a high increasing rate of strength gain over 30 MPa/day at one day and reached its highest value (44.74 MPa) after seven days, the sample deteriorated to half of the seven-day strength value by 28 days.

Many of the earlier studies [9,10,11,12], which observed similar strength deteriorations, concluded that the strength decline of hardened geopolymers with time were mainly due to shrinkage caused by the drying of pores during the curing processes; however, this explanation does not apply to the present study because the curing humidity condition was kept at more than 99% for this study. In addition, no dimensional shrinkage was observed in all hardened geopolymer samples during the moist curing.

**Table 3:** Results from the compressive strength testing.

Sample ID	Compressive strength (standard deviation), MPa		
	1-day	7-day	28-day
F-NW	32 (1)	44 (4)	24 (5)
FG2-NW	26 (2)	40 (2)	45 (5)
FG4-NW	19 (3)	25 (5)	30 (4)
FG6-NW	15 (3)	21 (3)	22 (2)

One possible cause of this strength deterioration in F-NW after seven days might be related to high Na concentration or the chemical composition of the raw fly ash (e.g., ~10% of Ca content). However, in a previous study [19], the same raw fly ash was alkali-activated using a 5M NaOH solution with no addition of Na-silicate, and no strength deterioration was observed until 28 days. Although different conditions for the alkali-activator were used in the previous study (e.g., a smaller NaOH concentration, a higher activator amount *etc*.), the activating solution supplied a higher quantity of Na^+^ ion moles per weight of raw fly ash (*i.e.*, 2.5 mole of Na^+^ per 1 kg of fly ash from NaOH) than the current study (*i.e.*, 2.2 mole of Na^+^ per 1 kg fly ash from NaOH and Na-silicate). Thus, the strength deterioration in this study was not merely associated to the Na concentration or the fly ash composition, but more likely related to the combined use of a high weight ratio of soluble silicate with a strong NaOH solution in alkali-activator [9].

The replacement of 2% to 6% fly ash with gypsum eliminated the strength deterioration observed in F-NW after seven days. The 2% replacement resulted in the best strength result, observed at 28 days (see Figure 1); however, the higher replacement ratios, more than 4%, markedly lowered the overall strength. Similar trends were observed in earlier studies that investigated the influence of gypsum with a range of replacement ratios for fly ash activated with NaOH and Na-silicate [4,8,13,14]. Those studies reported that the strengths of fly ash geopolymers increased up to a certain point of gypsum addition (~1% to 10%) and decreased above that addition; however, no detailed discussion concerning the causes of the strength decrease was made.

**Figure 2:**
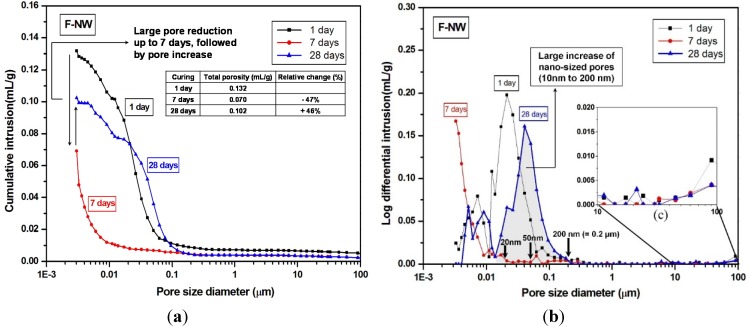
Pore size distribution curves of the F-NW samples (case of no gypsum addition) after one, seven, and 28 days of curing. (**a**) cumulative distribution; (**b**) log differential distribution; (**c**) a magnified plot for 10 to 100 μm pores, which are visible in the SEM images of the later section.

**Figure 3:**
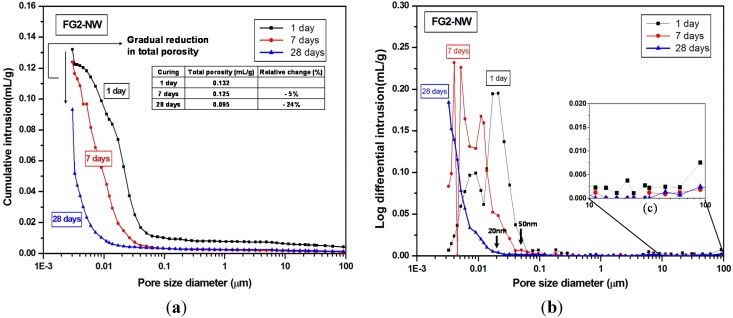
Pore size distribution curves of the FG2-NW samples (2% gypsum addition) after one, seven, and 28 days of curing. (**a**) cumulative distribution; (**b**) log differential distribution; (**c**) a magnified plot for 10 to 100 μm pores, which are visible in the SEM images of the later section.

Mercury intrusion porosimetry (MIP) testing, performed to compare F-NW and FG2-NW samples, illustrates how the use of gypsum affected the pore size distribution and strength deterioration, as presented in Figure 2 and Figure 3. In Figure 2a, the total porosity of F-NW (case of no gypsum addition) was notably reduced by seven days; however, the porosity remarkably increased by 46% at 28 days. Figure 2 illustrates a few important observations for F-NW: (1) at seven days, most of the pores were smaller than ~10 nm; (2) at 28 days, pores smaller than ~4 nm, which originally constituted ~67% of the total porosity at seven days, vanished; and (3) at 28 days, newly formed nano-sized pores from 10 to 200 nm represented ~73% of the total porosity. Accordingly, considering the high strength of the hardened matrix of F-NW at seven days (*i.e.*, 43.9 MPa), the increased porosity at 28 days indicates a large volume of nano-cracks, which were propagated from the nano-pores smaller than ~4 nm. Note that at least 30% of newly formed cracks are larger than ~50 nm. Because it is known that pores larger than ~50 nm largely reduce the compressive strength of hardened pastes [20], the extensive cracking with changes in the pore size and porosity should be responsible for the strength deterioration of F-NW after seven days. Moreover, geometrical shape of cracks is generally sharp rather than spherical and this makes the matrix more vulnerable to stress concentration.

Whereas, the total porosity of FG2-NW (sample with 2% gypsum) gradually decreased until 28 days, with no observed formation of a nano-cracking; the majority of pores at 28 days were smaller than 20 nm see (Figure 3a,b).

Note that the pore size distribution of FG2-NW at 28 days is similar to that of F-NW at seven days (see in-set tables in Figure 2 and Figure 3); this shows that the addition of gypsum delayed the evolution of pore structure over time, indirectly suggesting that the reaction rate was also slowed.

**Figure 4:**
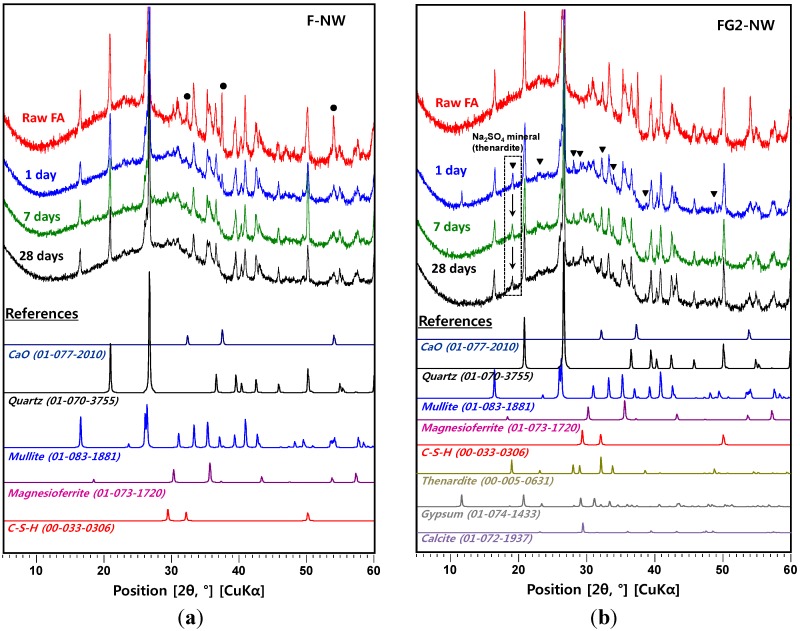

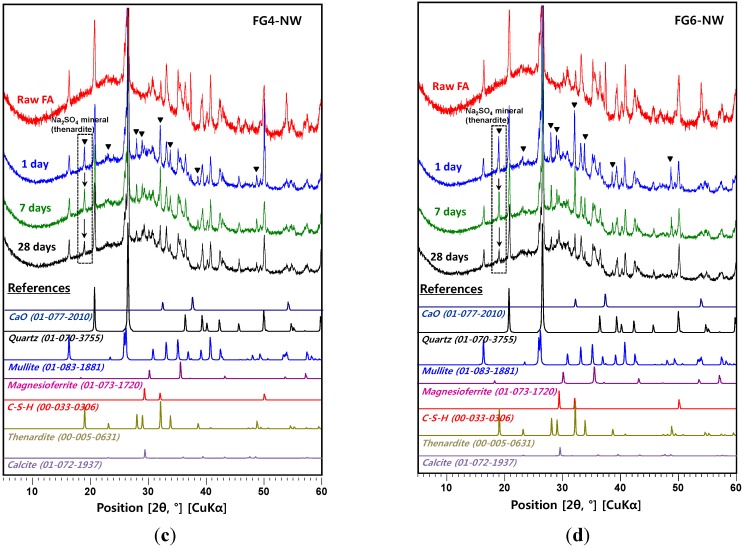
XRD patterns of samples by curing time. The simulated reference patterns (e.g., quartz, mullite) are scaled to have the same signal intensity observed in the one-day cured sample. The numbers in ( ) indicate ICDD PDF numbers. ●: peak positions of CaO. ▼: peak positions of thenardite. Raw fly ash is labeled as Raw FA. (**a**) F-NW sample; (**b**) FG2-NW sample; (**c**) FG4-NW sample; (**d**) FG6-NW sample.

**Figure 5:**
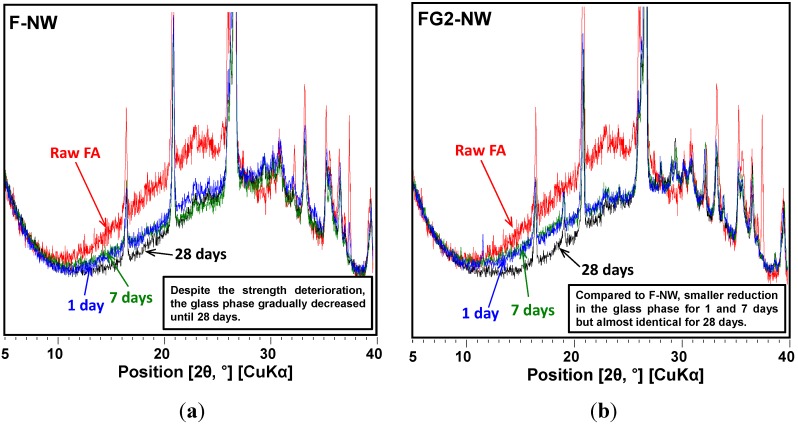

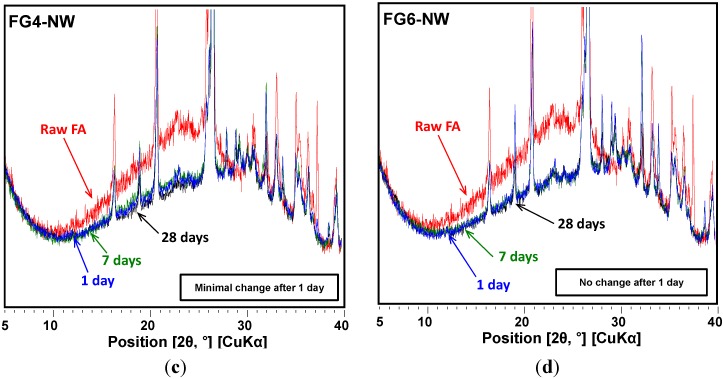
Progressive changes of overlapped XRD patterns for the amorphous phase (*i.e.*, 10° to 32°) as curing progresses. (**a**) F-NW sample; (**b**) FG2-NW sample; (**c**) FG4-NW sample; (**d**) FG6-NW sample.

Figure 4 and Figure 5 present the powder X-ray diffraction patterns for all of the hardened samples taken after curing for one, seven, and 28 days. Quartz, mullite, and magnesioferrite, which were originally contained in the raw fly ash, were identified in all samples. All of the activated samples for all curing ages showed amorphous humps centered around a 2θ of ~27° to 29°, indicating the formation of a geopolymeric gel [4,12,18]. No zeolite crystals were observed in any of the measured samples.

The raw fly ash consisted of 10.1 wt% of CaO in oxide composition (see the XRF result in Table 1) and some portion of this existed in a crystalline CaO, which is observed in the XRD pattern of raw fly ash (labeled Raw FA in figures). The CaO produced weak C-S-H peaks within one day after activation, with the complete loss of CaO peaks after seven days of curing. Although a few previous papers reported that calcium ions available from fly ash react with silicate ions to generate the C-S-H phase, resulting in strength increases [8,14], the C-S-H formation in the present study was likely not responsible for the observed strength gains because the sizes of the measured C-S-H peaks were comparatively small [19].

In a past study [12], the phase transformation of amorphous geopolymer into crystalline zeolitic phases with curing age was explained as one possible cause of strength deterioration in the geopolymer. In the present study, the formation of crystalline phases in F-NW was not observed after seven days; however, the mechanism proposed in the literature can explain the present study because there is a reasonable likelihood that the nano-cracking may be due to the fast growth of nano-crystalline zeolites. It is well known that geopolymer is composed of numerous nano-crystalline zeolites and aluminosilicate gel [18,21]. Readily available Si ions from Na-silicate may have accelerated the growth of high Si/Al zeolitic crystals (e.g., Na-chabazite) [7,22], seemingly exerting a destructive pressure on the hardened geopolymer matrix. If the present study used a prolonged curing period (e.g., 210 days), the nano-crystalline zeolites could grow into sizable zeolitic crystals, which can be detected by XRD, as observed in a previous study [12]. However, the crystals were still too small to be detected by the available XRD instrument in this study, even if the zeolitic crystals underwent fast growth [21].

The use of gypsum is likely to decrease the fast growth rate of the nano-crystalline zeolites. Because Na, Si, and Al are essential ingredients in zeolites, a temporary removal of any of these elements in the pore solution can delay zeolite growth. In the samples containing gypsum, formation of the crystalline phase of Na_2_SO_4_ (thenardite) was observed. Note that the solvent exchange method with drying process using a vacuum desiccator was used for all XRD sample preparations. Thus, mirabilite (Na_2_SO_4_·10H_2_O) [23], which is a hydrated form of thenardite, might be the original reaction product rather than thenardite because the severe drying process may have converted the mirabilite into thenardite. An increase in the quantity of gypsum leads to an increase in the amount of thenardite synthesized. Note that in each mixture, the quantity of thenardite gradually decreased with curing time (see the peak reductions of ▼ with time in Figure 4). This observation suggests that a considerable portion of Na ions were reserved by SO_4_^2−^, forming thenardite in the early stages of the reaction, and were continuously released by a dissolution process as curing progressed. The addition of 2% gypsum can provide SO_4_^2−^ ions up to ~0.12 moles per 1 kg of fly ash, resulting in an equivalent molar quantity of Na_2_SO_4_ production, which can retain ~0.24 moles of Na^+^ ions. Considering that the activating solution for F-NW produces ~2.2 moles of Na^+^ per 1 kg of fly ash, 2% gypsum addition can temporarily reduce the availability of Na^+^ by retaining up to ~11% Na^+^ in the form of thenardite. In the same way, 4% and 6% gypsum additions could reduce the Na availability by up to ~22% and ~33%, respectively, in the geopolymeric reaction. This explains why the samples with more than 4% gypsum developed noticeably low strength because the large amount of retained Na^+^ ions could not participate in the early stages of the geopolymeric reaction. However, under such strongly alkaline and dissolved silicate conditions (e.g., F-NW), a small quantity of Na^+^ reduction (e.g., ~11% as in FG2-NW) seems to be useful in preventing the strength deterioration, possibly due to a significantly slower growth of the nano-crystalline zeolites. More evidence will be discussed in the SEM results section.

Because gypsum is less soluble in water than Na_2_SO_4_ [24], SO_4_^2−^ should have a higher affinity for Ca^2+^ than Na^+^ in a precipitation reaction; however, no remaining gypsum was observed in any of the measured XRD patterns after seven days, even at the highest addition levels (*i.e.*, 6 wt%). The addition of gypsum produced only the Na_2_SO_4_ phase; this observation implies that Ca ions from gypsum was rapidly consumed even at low concentrations, producing Ca-containing phases, such as C-S-H or calcite (CaCO_3_), which were observed in the XRD patterns of the samples containing gypsum.

The progressive reduction of the amorphous hump between 10° and 32° observed in Figure 5 over the curing period demonstrates a gradual dissolution of the glass phase. Thus, Figure 5 provides useful information concerning the effect of gypsum addition on the glass phase dissolution as follows: (1) in F-NW, despite the strength deterioration, the glass phase was continuously reduced (or dissolved) up until the 28th day; (2) the 2% gypsum addition (*i.e.*, FG2-NW) reduced the dissolution rate of the glass phase up to the seventh day compared to the case of no gypsum (*i.e.*, F-NW); (3) at 28 days, the remaining glass content for F-NW and FG2-NW became almost identical, which implies the early dissolution process was only slowed when 2% gypsum was added; and (4) the excessive use of more than 4% gypsum terminated the dissolution process of the glass phase after one day (see FG4-NW and FG6-NW). Therefore, it may be concluded that the addition of gypsum not only affected the geopolymeric reaction by reserving Na ions but also delayed the glass dissolution process, which is a Si provider. When a critical amount of gypsum is used, the strength deterioration during the curing process may be prevented by temporarily reducing the excessive Si concentration. Because the glass dissolution is predominantly governed by the availability of OH^-^ in alkali-activation [25,26], the gypsum addition probably affected the pH of the pore solution; however, a detailed mechanism is not clearly identified in this study, and requires further investigation.

**Figure 6:**
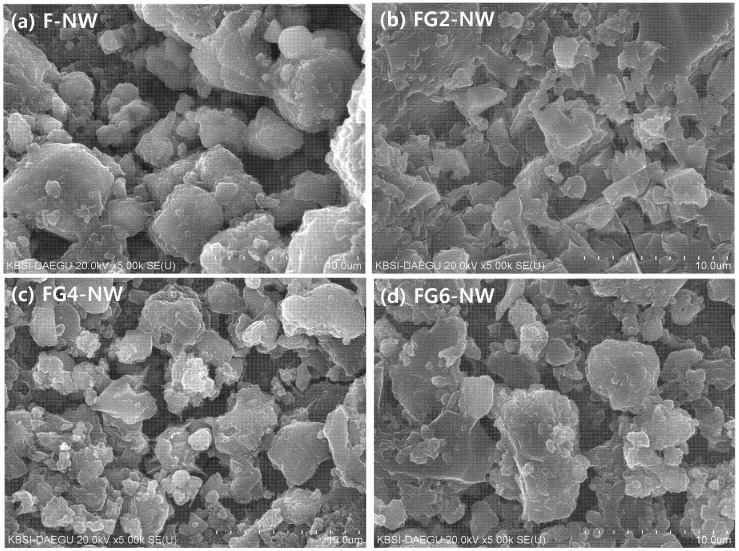
Microstructure of samples measured at 28 days by SEM in SE image mode. (**a**) F-NW sample (case of no gypsum addition); (**b**) FG2-NW sample (sample with 2% gypsum); (**c**) FG4-NW sample (sample with 4% gypsum); (**d**) FG6-NW sample (sample with 6% gypsum).

**Figure 7:**
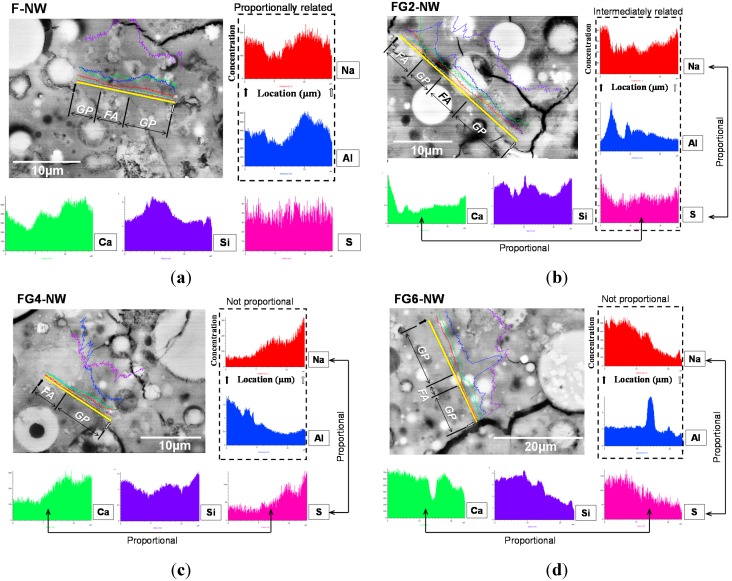
Elemental line scanning results for the hardened matrices. The location of the line scan is indicated with a thick straight line in each BSE image. GP: geopolymer phase and FA: fly ash particle: (**a**) F-NW sample (case of no gypsum addition); (**b**) FG2-NW sample (sample with 2% gypsum); (**c**) FG4-NW sample (sample with 4% gypsum); (**d**) FG6-NW sample (sample with 6% gypsum).

**Figure 8:**
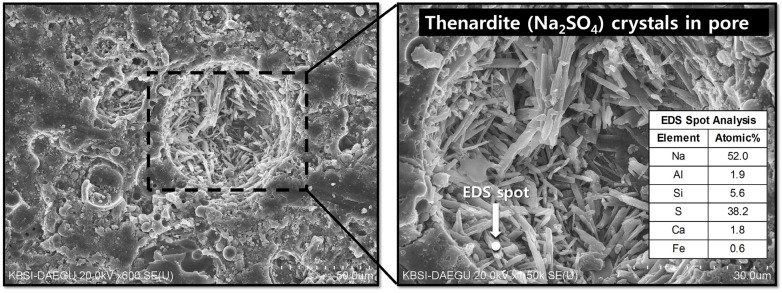
Crystals of thenardite (Na_2_SO_4_) observed in a pore space of FG6-NW (sample with 6% gypsum) at 28 days.

Figure 6 and Figure 7 show representative pore areas in the SE imaging mode and polished cross-sections of hardened matrices in the BSE imaging mode, respectively, at 28 days. The visible cracks in BSE images are similarly found in the literature [27,28]. All of the measured samples show minimal microstructural differences despite the large strength differences. In particular, it is not surprising that the widespread cracks that developed in F-NW after seven days (see Figure 2b) are not visible in the BSE images because most of the cracks were found to be on the order of 10 s of nanometers, as determined by the MIP results. However, the results from the elemental line scans, as shown in Figure 7, illustrate clear differences between the samples; for each sample, 10 times of elemental line scans were carried out at arbitrarily chosen locations on the sample and the representative line scanning results of this study are illustrated in Figure 7. When no gypsum was used (F-NW), there was an apparent proportional relationship between Na and Al (see Figure 7a), which is a necessary condition for geopolymer formation [29]. With an increasing amount of gypsum, the concentration of Na was no longer proportional to that of Al (see Figure 7b–d), implying that geopolymer formation was probably hindered. Moreover, with an increase in the gypsumquantity, Na became more proportionally associated with S, which supports the earlier conclusion that a high portion of Na^+^ was absorbed by SO_4_^2−^ in the form of Na_2_SO_4_. Thus, the line scan results in Figure 7 may suggest one possibility that most of Na_2_SO_4_ are probably present in pores because thenardite crystals were often observed in pore spaces for FG6-NW (see Figure 8); because the MIP result shows that FG2-NW contains mostly nanometer-sized pores at 28 days, a high portion of Na ions could be trapped in the nanometer-sized pores in the form of Na_2_SO_4_. It is also worth noting that Ca was still related to S, as shown in Figure 7b–d, because a considerable portion of Ca was released due to the addition of gypsum.

## 4. Conclusions

This study discussed the role of Na_2_SO_4_, synthesized from the addition of CaSO_4_·H_2_O, as a sodium reservoir in the formation of a Class F fly ash based geopolymer under highly alkaline silicate solution conditions based on the results of compressive strength testing, MIP, XRD, and SEM (SE/BSE/EDS) for the characterization of the material properties. This work resulted in the following experimental conclusions:

1. When Class F fly ash is activated with a highly alkaline silicate solution under an elevated temperature (*i.e.*, F-NW), the hardened matrix has a chance to show significant strength deterioration with increased curing time.
2. The strength deterioration is not solely associated with the high Na concentration or the composition of the fly ash but is more likely related to a high simultaneous concentration of Na and Si ions at an early stage of geopolymer formation.
3. The replacement of fly ash with 2% to 6% gypsum eliminated the strength deterioration; the 2% replacement resulted in the best strength value at 28 days; however, at higher replacement ratios, greater than 4%, a markedly lowered overall strength was observed.
4. The MIP result identified a large volume of increased porosity in F-NW after seven days, indicating extensive nano-sized crack formation (~10 to 200 nm), which is related to strength deterioration; moreover, the sample with 2% gypsum did not show any evidence of similar crack formation.
5. The use of gypsum obviously delayed the evolution of pore structures over time, indirectly suggesting that the rate of the geopolymeric reaction was also slowed.
6. In the XRD results, no observable crystalline phase was directly related to the strength deterioration and nano-sized cracking. The cracking was likely due to the fast growth of the nano-crystalline zeolitic phase, exerting a destructive pressure on the hardened matrix. The presence of soluble silicate largely promoted the growth rate of the zeolitic crystals; however, the sizes of the crystals formed over the 28-day curing interval were too small to be detected by XRD.
7. In the samples with gypsum, a crystalline phase of Na_2_SO_4_ (thenardite) formed, suggesting that a considerable portion of Na ions were reserved by SO_4_^2−^, widely forming thenardite in nanometer-sized pores at the early stages of the reaction, and were continuously released by dissolution processes as curing progresses. Thus, the addition of 2% gypsum can temporarily reduce the availability of Na^+^, up to ~11%, in the matrix. The small quantity of Na^+^ reduction appears to assist in preventing the strength deterioration, possibly due to a significant reduction in the growth rate of zeolitic crystals.
8. The gypsum addition not only affected the geopolymeric reaction by reserving Na ions, but also delayed the glass dissolution process, which is a Si provider. When a proper amount of gypsum was used, the strength deterioration might have been prevented by temporarily reducing excessive Si concentration during geopolymer formation.
9. The elemental line scanning results of the SEM work illustrated that the synthesized Na_2_SO_4_ phase was widely dispersed in the geopolymer, implying extensive reservation of Na^+^ by SO_4_^2^^−^; when gypsum addition was excessive, a substantial amount of Na ions were eliminated without participating in the geopolymeric reaction.
